# Investigation on the Coordination Bonding Nature of Actinide-Doped Endohedral Borospherenes An@B_40_^0/+/−^ (An = U, Np, Pu, Am, Cm)

**DOI:** 10.3390/molecules29245879

**Published:** 2024-12-13

**Authors:** Xiao-Ni Zhao, Zhi-Hong Wei, Si-Dian Li

**Affiliations:** Key Laboratory of Chemical Biology and Molecular Engineering of Education Ministry, Institute of Molecular Science, Shanxi University, Taiyuan 030006, China; zhaoxiaoni@sxu.edu.cn (X.-N.Z.); weizhihong@sxu.edu.cn (Z.-H.W.)

**Keywords:** actinides, metallo-borospherenes, first-principles theory, structures, coordination bonding patterns

## Abstract

Endohedral metallo-borospherenes M@B_40_ have received considerable attention since the discovery of B_40_ in 2014. However, the coordination bonding nature of most of actinide-doped endohedral An@B_40_ still remains in dispute or unexplored. Extensive and systematic first-principles theory calculations performed herein unveil the ground states of triplet U@B_40_ (**1**, *C*_2*v*_, ^3^A_2_), quartet U@B_40_^−^ (**2**, *C*_2*v*_, ^4^B_1_), quintet Np@B_40_^+^ (**3**, *C*_2*v*_, ^5^A_1_), sextet Np@B_40_ (**4**, *C*_2_, ^6^A), septet Pu@B_40_ (**5**, *C*_2*v*_, ^7^A_2_), octet Am@B_40_ (**6**, *C*_2*v*_, ^8^A_2_), and octet Cm@B_40_^+^ (**7**, *C*_2*v*_, ^8^A_2_) at the coupled-cluster with triple excitations CCSD(T) level. Detailed principal interacting spin orbital (PISO) and adaptive natural density partitioning (AdNDP) analyses reveal their coordination bonding patterns and show that, with the numbers of unpaired *α*-electrons in parallel spins varying from n_α_ = 2, 3, 4, 5, 6, 7, to 7 in these complexes, the percentage contribution of the An 5f-involved PISO pairs to overall coordination bonding interactions decreases monotonously from 41% to 1%, and the contribution of An 6d-involved PISO pairs increases monotonously from 47% to 72%, while the marginal contribution of An 7s-involved PISO pairs remains basically unchanged (4~7%). The IR, Raman, and photoelectron spectra of the most concerned species are computationally simulated to facilitate their characterizations in future experiments.

## 1. Introduction

The discovery of the first all-boron fullerenes *D*_2*d*_ B_40_^−/0^ in 2014 [1] and *C*_3_/*C*_2_ B_39_^−^ in 2015 [2] paved the way for borospherene chemistry [3]. Seashell-like B_28_^−/0^ [4] and bilayer B_48_^−/0^ [5] were experimentally observed later to further expand the borospherene family [6,7,8]. Considerable attention has been paid to the structures and bonding of their metal-doped endohedral and exohedral metallo-borospherenes in the past decade. Our group predicted at a density functional theory (DFT) level the first endohedral metallo-borospherenes *C*_2*v*_ Ca@B_40_ and *D*_2*d*_ Sr@B_40_ and exohedral metallo-borospherene *C_s_* M&B_40_ (M = Be, Mg) in 2015 [9]. Similar endohedral rare-earth-metal-doped *C_s_* Sc@B_40_, *C*_2*v*_ Y@B_40_, and *C*_2*v*_ La@B_40_ have also been proposed by DFT [10]. Dong et al. proposed a B_40_ fullerene decorated with six Ti atoms as a promising candidate for hydrogen storage [11]. Fa et al. studied the structural stability of endohedral *C*_2*v*_ Na@B_40_ and *D*_2*d*_ Ba@B_40_ and exohedral *C_s_* M&B_40_ (M = Li, K and Tl) by DFT [12]. Sr doping was found to increase the conductance of B_40_ fullerene due to the decreased energy gap in *D*_2*d*_ Sr@B_40_ [13]. The Ti atom in Ti@B_40_ is found to reside very close to the boron framework, while the doubly doped Ti_2_@B_40_ possesses a singlet cube-like structure with *C_s_* symmetry [14]. The exohedral Ni*_n_*∈B_40_ complex series (n = 1–4) features quasi-planar hepta-coordinate Ni centers on the cage surfaces in *η*^7^-B_7_ heptagons [15]. Li et al. predicted that Cu, Ag, and Au atoms in MB_40_ (M = Cu, Ag, and Au) favor the exohedral configuration [16]. Wang et al. predicted in 2017 the first singlet endohedral actinide-metal-doped *D*_2*d*_ U@B_40_ at a pure DFT Perdew–Burke–Ernzerhof (DFT-PBE) level which, with the U atom located exactly at the center of the B_40_ cage, satisfies the 32-electron principle of 1S^2^1P^6^1D^10^1F^14^ [17]. However, at the hybrid PBE0 level, a slightly distorted triplet *C*_1_ U@B_40_ was found to be the ground state of the magnetic neutral species, which is 0.70 eV more stable than its singlet counterpart *D*_2*d*_ U@B_40_ [18]. Shi et al. explored an actinide-doped AnB*_m_* series (An = Ac, Th, Pa, U, Np, Pu, Am, Cm; *m* = 7, 20, 24, 36, 38, 39, 40) and suggested that doping with the right actinides may stabilize B*_n_* clusters [19,20,21,22]. Octet lanthanide-doped *D*_2*d*_ Eu@B_40_ (^8^B_2_) and septet *C_s_* Gd@B_40_ (^7^A′′) have also been predicted in theory [23]. Li et al. explored Th@B_40_, which revealed obvious covalent characters between the Th center and the B_40_ cage [24]. However, the coordination bonding nature of most of the actinide-doped endohedral An@B_40_ complexes, especially how the 5f, 6d, and 7s valence orbitals of the An coordination centers participate in the coordination bonding patterns of the systems and how their contributions to the overall coordination bonding interactions evolve in the An@B_40_ series, still remains unknown or in dispute in the literature.

In this work, we systematically investigated the coordination bonding nature of actinide-doped endohedral borospherenes An@B_40_^0/+/−^ (An = U, Np, Pu, Am, Cm) at the first-principles theory level, aiming to clarify the situation in the complex series. Extensive coupled-cluster calculations with triple excitations (CCSD(T)) reveal the ground states of U@B_40_ (**1**, *C*_2*v*_, ^3^A_2_), U@B_40_^−^ (**2**, *C*_2*v*_, ^4^B_1_), Np@B_40_^+^ (**3**, *C*_2*v*_, ^5^A_1_), Np@B_40_ (**4**, *C*_2_, ^6^A), Pu@B_40_ (**5**, *C*_2*v*_, ^7^A_2_), Am@B_40_ (**6**, *C*_2*v*_, ^8^A_2_), and Cm@B_40_^+^ (**7**, *C*_2*v*_, ^8^A_2_) with the numbers of unpaired α-electrons of n_α_ = 2, 3, 4, 5, 6, 7, and 7, respectively. Detailed principal interacting spin orbital (PISO) and adaptive natural density partitioning (AdNDP) analyses unveil the coordination bonding patterns of the complex series and quantitatively evaluate the variation trends of percentage contributions of An 5f-, 6d-, and 7s-involved PISO pairs to the overall coordination bonding energies with the numbers of unpaired α-electrons (n*_α_*) in the complex systems.

## 2. Results and Discussion

### 2.1. Structures and Stabilities

**Figure 1:**
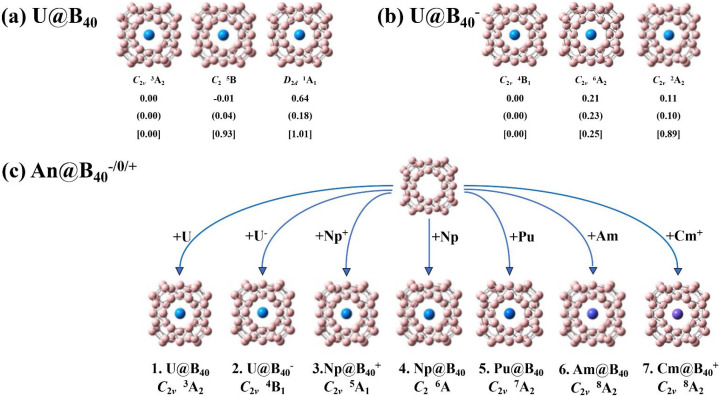
Three optimized low-lying isomers of (**a**) U@B_40_ and (**b**) U@B_40_^−^ with their relative energies indicated in eV at PBE0, TPSSh (parentheses), and CCSD(T)/PBE0 (square brackets) levels, respectively, and optimized ground-state structures of (**c**) *C*_2*v*_ U@B_40_ (**1**,^3^A_2_), *C*_2*v*_ U@B_40_^−^ (**2**, ^4^B_1_), *C*_2*v*_ Np@B_40_^+^ (**3**, ^5^A_1_), *C*_2_ Np@B_40_ (**4**, ^6^A), *C*_2*v*_ Pu@B_40_ (**5**, ^7^A_2_), *C*_2*v*_ Am@B_40_ (**6**, ^8^A_2_), and *C*_2*v*_ Cm@B_40_^+^ (**7**, ^8^A_2_) at the PBE0 level.

The optimized three lowest-lying isomers with different spin multiplicities of U@B_40_ and U@B_40_^−^ are shown in Figure 1a and Figure 1b, respectively. The optimized ground-state structures of the An@B_40_^0/+/−^ series (An = U, Np, Pu, Am, Cm) are collectively shown in Figure 1c, with their alternative low-lying isomers with different spin multiplicities depicted according to their relative energies in Figure S1. As shown in *Figure 1 and Figure S1*, the calculated CCSD(T) relative energies at the most accurate theoretical level implemented in this work provide strong evidence to support both the hybrid PBE0 and TPSSh approaches.

Interestingly, as clearly shown in Figure 1a, the triplet *C*_2*v*_ U@B_40_ (**1**, ^3^A_2_) with two unpaired 5f α-electrons proves to be the well-defined ground state of the neutral complex, which is 0.93 and 1.01 eV more stable than the quintet *C*_2_ U@B_40_ (^5^B) and singlet *D*_2*d*_ U@B_40_ (^1^A_1_) at the most accurate CCSD(T) level achieved in this work, respectively. Such a relative energy order qualitatively agrees with that obtained at both the hybrid DFT-PBE0 and DFT-TPSSh levels but totally differs from that of previously reported results at a pure DFT-PBE level [18]. We believe the hybrid DFT and CCSD(T) relative energies are more reliable than that obtained at the pure DFT-PBE level. As expected, with one extra electron attached, the U@B_40_^−^ monoanion with three unpaired 5f α-electrons appears to have a quartet ground state of *C*_2*v*_ U@B_40_^−^ (**2**, ^4^B_1_), which is 0.25 and 0.89 eV more stable than the sextet *C*_2*v*_ U@B_40_^−^ (^6^A_2_) and doublet *C*_2*v*_ U@B_40_^−^ (^2^A_2_) at the CCSD(T) level, respectively. Detailed BOMD simulations collectively shown in Figure S2 indicate that both U@B_40_ (**1**) and U@B_40_^−^ (**2**) are dynamically stable at 300 K, with small calculated root-mean-square-deviations (RMSDs) of 0.12, 0.08 Å and maximum bond length deviations (MAXDs) of 0.41, 27 Å, respectively.

By substituting the U coordination center in U@B_40_ (**1**) with heavier actinide metals Np, Pu, Am, and Cm, the quintet *C*_2*v*_ Np@B_40_^+^ (**3**, ^5^A_1_), sextet *C*_2_ Np@B_40_ (**4**, ^6^A), septet *C*_2*v*_ Pu@B_40_ (**5**, ^7^A_2_), octet *C*_2*v*_ Am@B_40_ (**6**, ^8^A_2_), and *C*_2*v*_ Cm@B_40_^+^ (**7**, ^8^A_2_) are obtained systematically, which prove to be the ground states of the systems as shown in Figure 1c and Figure S1, with the second lowest-lying *C*_1_ Np@B_40_^+^ (^7^A), *C*_2*v*_ Np@B_40_ (^4^B_2_), *C*_2*v*_ Pu@B_40_ (^5^B_2_), *C*_2*v*_ Am@B_40_ (^10^B_1_), and *C*_s_ Cm@B_40_^+^ (^6^A′) being 0.41, 0.19, 0.07, 0.04, and 0.77 eV less stable than their corresponding ground states at the CCSD(T) level, respectively. As discussed in detail below, the ground states triplet U@B_40_ (**1**), quartet U@B_40_^−^ (**2**), quintet Np@B_40_^+^ (**3**), sextet Np@B_40_ (**4**), septet Pu@B_40_ (**5**), octet Am@B_40_ (**6**), and octet Cm@B_40_^+^ (**7**) possess increasing spin multiplicities, with the numbers of unpaired α-electrons in parallel spins varying from n_α_ = 2, 3, 4, 5, 6, 7, to 7, indicating that the increased valence electrons in complexes **1**–**7** are consecutively distributed in unpaired α-orbitals of the systems.

### 2.2. Bonding Pattern Analyses

**Figure 2:**
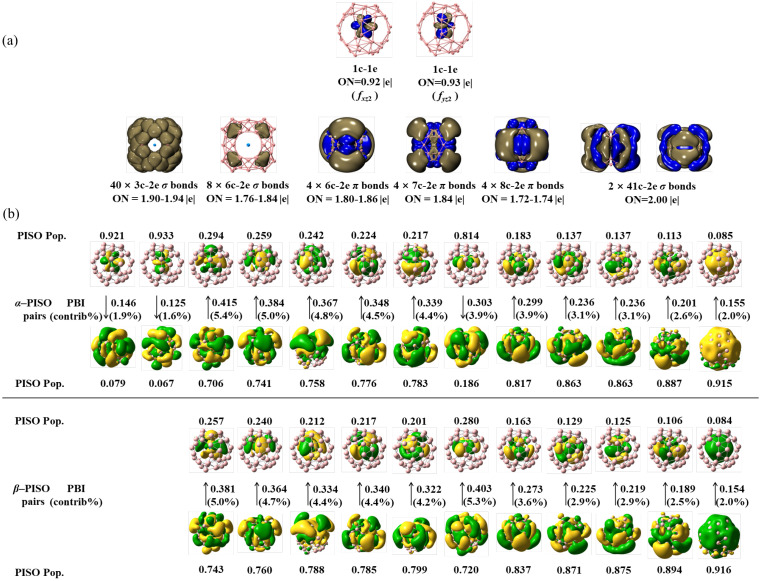
(**a**) AdNDP bonding pattern of triplet *C*_2*v*_ U@B_40_ (**1**), with the occupation numbers (ON) indicated. (**b**) PISO bonding pattern of *C*_2*v*_ U@B_40_ (**1**) with the U coordination center and B_40_ ligand as interacting fragments, with the corresponding occupation numbers (PISO Pop.), PIO-based bond indexes (PBI), and percentage contributions (contrib/%) to the overall coordination interactions indicated.

As demonstrated, detailed AdNDP and PISO bonding patterns of the triplet *C*_2*v*_ U@B_40_ (**1**) are presented in Figure 2. As shown in Figure 2a, U@B_40_ (**1**) contains one 1c–1e *f_xz_*_2_-type bond and one 1c–1e *f_yz_*_2_-type bond on the U coordination center with occupation numbers of ON = 0.92 and 0.93, respectively, forty 3c–2e and eight 6c–2e σ bonds on the B_40_ ligand with ON = 1.76–1.94, four 6c–2e, four 7c–2e, and four 8c–2e π coordination bonds between the B_40_ ligand and U center with ON = 1.72–1.86, and two 41c–2e σ coordination bonds between B_40_ and U with ON = 2.00. It is the two unpaired 5f α-electrons in parallel spins that determine the triplet ground state of the system (^3^A_2_).

Detailed PISO analyses on U@B_40_ (**1**) in Figure 2b with the B_40_ ligand and U coordination center as interacting fragments help to unveil a precise description of the coordination bonding pattern in the complex. As clearly shown in Figure 2b, U@B_40_ (**1**) has two unpaired α-PISO 5f orbitals with the PISO populations of 0.921 and 0.933, respectively, as the singly occupied molecular orbitals (SOMOs) of the complex, which have no corresponding β-PISO counterparts to correlate with, while all the remaining α-PISO and β-PISO pairs in exact one-to-one corresponding relationships are fully paired in couples, rendering the system a triplet ground state (^3^A_2_). The two unpaired α-PISO 5f orbitals turn out to correspond well to the 1c–1e *f_xz_*_2_-type bond and 1c–1e *f_yz_*_2_-type bond obtained by AdNDP analyses in Figure 2a, respectively. Their small PISO-based bond indexes (PBI) of 0.146 and 0.125 indicate that the interactions between the two unpaired α-PISO U 5f orbitals and the corresponding nearly empty α-molecular orbitals of the B_40_ ligand with the small PISO populations of 0.079 and 0.067 make marginable contributions (1.9% and 1.6%, respectively) to the overall coordination energy in the complex, with the remaining PISO pairs with PISO 5f populations between 0.084 and 0.294, and PBI values between 0.154 and 0.415 dominate the overall coordination interactions between the B_40_ ligand and U center. In combinations, these PISO pairs result in four 6c–2e, four 7c–2e, and four 8c–2e π coordination bonds, and the two 41c–2e σ coordination bonds obtained in the AdNDP analyses discussed above.

**Figure 3:**
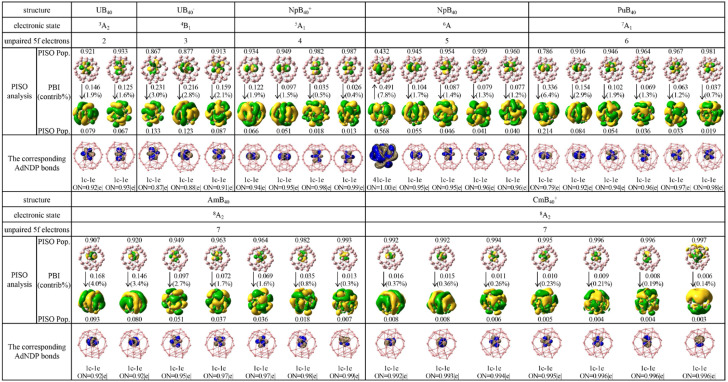
Unpaired PISO α-orbitals of U@B_40_ (**1**, ^3^A_2_), U@B_40_^−^ (**2**, ^4^B_1_), Np@B_40_^+^ (**3**, ^5^A_1_), Np@B_40_ (**4**, ^6^A), Pu@B_40_ (**5**, ^7^A_2_), Am@B_40_ (**6**, ^8^A_2_), and Cm@B_40_^+^ (**7**, ^8^A_2_), with the α-spin occupation numbers (PISO Pop.) associated with the principal interacting spin orbitals, PISO-based bond indexes (PBI), and their percentage contributions (contrib/%) to the overall coordination interactions between the An coordination center and B_40_ ligand indicated. The corresponding AdNDP analyses of the singly occupied 1c–1e α-5f orbitals in **1**, **2**, **3**, **5**, **6**, and **7** and 41c–1e α-bond in Np@B_40_ (**4**) are compared at the bottom, with the occupation numbers (ON) indicated.

As shown in Figure 3, similar AdNDP and PISO bonding patterns exist for quartet U@B_40_^−^ (**2**, ^4^B_1_), quintet Np@B_40_^+^ (**3**, ^5^A_1_), septet Pu@B_40_ (**5**, ^7^A_2_), octet *C*_2*v*_ Am@B_40_ (**6**, ^8^A_2_), and octet *C*_2*v*_ Cm@B_40_^+^ (**7**, ^8^A_2_), which possess three, four, six, seven, and seven unpaired α-PISO 5f electrons with the PISO populations between 0.87 and 0.91, 0.93 and 0.99, 0.79 and 0.98, 0.91 and 0.99, and 0.99 and 1.00, and the following PISO-based bond indexes: PBI = 0.16~0.23, 0.03~0.12, 0.04~0.34, 0.01~0.17, 0.01~0.02, respectively. Interestingly, the α-SOMO of the sextet Np@B_40_ (**4**), which contributes 7.8% to the overall coordination interaction, turns out to be a typical α-bond with a comparable Np 5f α-PISO population of 0.432 and B_40_ β-PISO population of 0.568, and a PISO-based bond index of PBI = 0.491. Such an α-bond with non-negligible contributions from both the Np coordination center and B_40_ ligand corresponds to a 41c–1e bond in AdNDP bonding analyses as clearly shown in Figure 3.

### 2.3. Percentage Contributions of An 5f-, 6d-, and 7s-Involved PISO Pairs to the Overall Coordination Interactions

**Figure 4:**
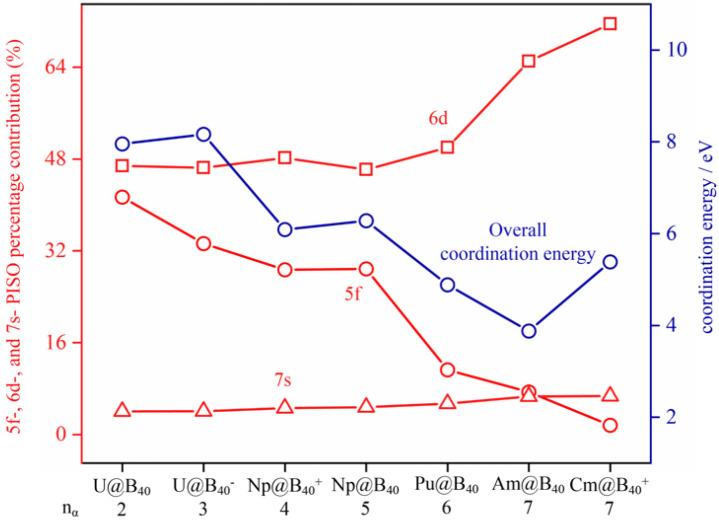
Variation of the calculated overall An--B_40_ coordination interaction energies highlighted in blue and the corresponding PISO percentage contributions of 5f-, 6d-, and 7s-orbital-involved pair interactions highlighted in red in U@B_40_ (**1**, ^3^A_2_), U@B_40_^−^ (**2**, ^4^B_1_), Np@B_40_^+^ (**3**, ^5^A_1_), Np@B_40_ (**4**, ^6^A), Pu@B_40_ (**5**, ^7^A_2_), Am@B_40_ (**6**, ^8^A_2_), and Cm@B_40_^+^ (**7**, ^8^A_2_) with the numbers of singly occupied 5f electrons (n_α_) at the PBE0 level.

To compare the percentage contributions of An 5f-, 6d-, and 7s-involved PISO pairs to the overall An--B_40_ coordination interaction energies, we categorized the orbital types of An atoms involved in the PISO bonding patterns by their orbital shapes and considered the contributions of the corresponding PISO pairs separately. As shown in Figure 4, with the numbers of unpaired α-electrons in parallel spins varying from n_α_ = 2, 3, 4, 5, 6, 7, to 7 in the complex series, the calculated overall An--B_40_ coordination interaction energies decrease generally from U@B_40_ (**1**, ^3^A_2_), U@B_40_^−^ (**2**, ^4^B_1_), Np@B_40_^+^ (**3**, ^5^A_1_), Np@B_40_ (**4**, ^6^A), Pu@B_40_ (**5**, ^7^A_2_), to Am@B_40_ (**6**, ^8^A_2_), and increase slightly at Cm@B_40_^+^ (**7**, ^8^A_2_), with the percentage contributions of An 5f-involved PISO pairs to the overall coordination bonding interactions decreasing monotonously from 41% to 1%, the dominating contributions of An 6d-involved PISO pairs increasing monotonously from 47% to 72%, and the marginal contributions of An 7s-involved PISO pairs remaining basically unchanged (4~7%). As major contributors in specific examples, in the much-concerned triplet *C_2v_* U@B_40_, the 5f-, 6d-, and 7s-involved PISO pairs contribute 3.29, 3.72, and 0.32 eV to the overall coordination interaction energy of 7.95 eV of the complex, respectively, while in octet *C*_2*v*_ Am@B_40_, the 5f-, 6d-, and 7s-involved PISO pairs contribute 0.29, 2.52, and 0.26 eV to the overall coordination interaction energy of 3.88 eV, respectively. The slight increase in overall coordination interaction energy at Cm@B_40_^+^ (**7**) mainly originates from the obvious increased contribution of the Cm 6d orbitals. These results show that with the metal center varying from U, Np, Pu, Am, to Cm, the tendency of the An-5f orbitals to participate in coordination bonding interactions with the B_40_ ligand weakens gradually from left to right in the periodic table, with the seven unpaired 5f α-electrons (5f^7^) in Cm@B_40_^+^ (**7**) contributing only about 1% to the overall coordination interaction energy, indicating an obvious actinide contraction in atomic radii from left to right in the periodic table. Figure 4 indicates that the An 6d atomic orbitals dominate the coordination interaction between the An centers and B_40_ ligand in the concerned An@B_40_ species, while An 5f and 7s make only minor contributions.

### 2.4. Simulated IR, Raman, and PE Spectra

**Figure 5:**
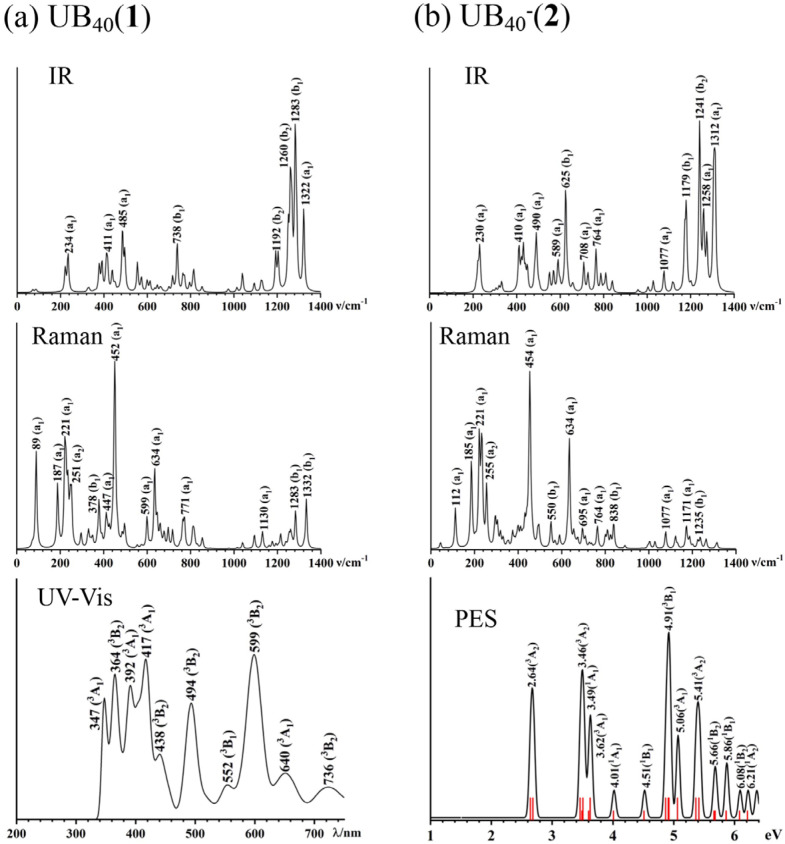
Simulated IR, Raman, and UV–Vis spectra of (**a**) *C*_2*v*_ U@B_40_ (**1**, ^3^A_2_), and IR, Raman, and photoelectron spectra (PES) of (**b**) *C*_2*v*_ U@B_40_^−^ (**2**, ^4^B_1_) at the PBE0 level.

Infrared (IR) and photoelectron spectra (PES) measurements have proven to be powerful approaches to characterize boron nanoclusters in gas phases [1,2,3]. We depict the simulated IR, Raman, and UV–Vis spectra of U@B_40_ (**1**) in Figure 5a and the calculated IR, Raman, and PES of U@B_40_^−^ (**2**) in Figure 5b at the PBE0 level to facilitate their future experimental characterizations. U@B_40_ (**1**) exhibits strong IR peaks at 234 (a_1_), 411 (a_1_), 485 (a_1_), 738 (b_1_), and 1283 (b_1_) cm^−1^, while its Raman spectrum features strong vibrational modes at 89 (a_1_), 221 (a_1_), 452 (a_1_), 634 (a_1_), and 1332 (b_1_). It is noticed that U@B_40_ (**1**) and U@B_40_^−^ (**2**) possess radial breathing modes (RBMs) at 452 cm^−1^ (a_1_) and 454 cm^−1^ (a^1^), respectively, which turn out to be slightly blue-shifted from that (428 cm^−1^ (a_1_)) of the empty *D*_2*d*_ B_40_ borospherene at the same theoretical level. Similar IR and Raman spectra exist for U@B_40_^−^ (**2**). The UV–Vis spectrum of U@B_40_ (**1**) and PES spectrum of U@B_40_^−^ (**2**) were calculated using a time-dependent DFT approach (TD-DFT) at the PBE0 level. Since U@B_40_^−^ has a quartet state, one electron detachment from the anion could lead to triplet or singlet final states in the neutral. The first vertical detachment energy at VDE1 = 2.64 eV (^3^A_2_) for U@B_40_^−^ was calculated as the energy difference between the anionic ground state and the neutral ground state at the optimized anion geometry. Higher vertical detachment energies at VDE = 3.46 (^3^A_2_), 4.91 (^3^B_1_), and 5.41 (^3^A_2_) eV correspond to vertical detachment transitions to the excited states of the neutral.

## 3. Theoretical Methods

The structures of endohedral actinide-metal-doped An@B_40_^0/+/−^ (An = U, Np, Pu, Am, Cm) were fully optimized at both hybrid DFT-PBE0 [25] and DFT-TPSSh [26] levels, with the 6-311+G(d) [27] basis set used for B and the scalar-relativistic Stuttgart energy-consistent pseudopotential with the 32-valence-electron and associated ECP60MWB_SEG valence basis set [28,29] employed for An. Single-point relative energies were further refined with the more accurate domain-based local pair-natural orbital-based singles and doubles-coupled cluster method (DLPNO-CCSD(T)) [30] implemented in the ORCA 5.0.3 package [31], with the segmented all-electron relativistically contracted basis sets with DKH2 Hamiltonians (SARC-DHK-TZVP) used for An and the DKH-def2-SVP basis set chosen for B [32]. Vibrational frequency and wavefunction stability checks were performed at the PBE0 level to make sure that all the lowest-lying isomers obtained were true minima of the systems without imaginary frequencies. All the PBE0 and TPSSh computations were performed using the Gaussian 09 [33] program package. Detailed Born–Oppenheimer molecular dynamic (BOMD) simulations were performed on both U@B_40_ (**1**) and U@B_40_^−^ (**2**) at 300 K for 30 ps. BOMD simulation was implemented employing CP2K [34] code with GTH-PBE pseudopotentials and TZVP-MOLOPTSR-GTH basis sets. The infrared and Raman spectra of *C*_2*v*_ U@B_40_ (**1**) and *C*_2*v*_ U@B_40_^−^ (**2**) were simulated at PBE0/6-311+G(d). The UV–vis absorption spectra of U@B_40_ (**1**) and PE spectrum of U@B_40_^−^ (**2**) were simulated using the time-dependent DFT method (TD-DFT-PBE0) approach [35,36].

Chemical bonding patterns were analyzed, employing both the AdNDP [37,38] method and principal interacting orbital (PIO) [39] approach based on the natural population analyses using the NBO 6.0 [40] program. In this work, PISO [41] analyses based on PIO calculations were performed on the open-shell An@B_40_^−/0/+^ series. The PIO analyses were also carried out using the Gaussian 09 program with the 6-31G* basis set used for B atoms and ECP60MWB_SEG employed for An. The VMD [42] program was used for the visualization of structures and molecular orbitals (https://www.ks.uiuc.edu/Research/vmd/, accessed on 27 October 2024).

## 4. Conclusions

In summary, we have predicted in this work the ground states of triplet U@B_40_ (**1**), quartet U@B_40_^−^ (**2**), quintet Np@B_40_^+^ (**3**), sextet Np@B_40_ (**4**), septet Pu@B_40_ (**5**), octet Am@B_40_ (**6**), and octet Cm@B_40_^+^ (**7**) at the CCSD(T) level, revealed their coordination bonding patterns using both the PISO and AdNDP approaches, and calculated the percentage contributions of An 5f-, 6d-, and 7s-involved PISO pairs to the overall coordination interaction energies at the PBE0 level, unveiling the coordination bonding nature of these actinide-doped endohedral metallo-borospherenes both qualitatively and quantitatively. Such high spin-multiplicity actinide-doped endohedral metallo-borospherenes could be extended to all the actinides in the periodic table to form various nanoclusters and crystals, which may serve as potential magnetic materials.

## Data Availability

The original contributions presented in the study are included in the article/Supplementary Materials, further inquiries can be directed to the corresponding author.

## Supplementary Materials

The following supporting information can be downloaded at https://www.mdpi.com/article/10.3390/molecules29245879/s1, Figure S1: Relative energies of the low-lying isomers of An@B_40_^0/+/−^ (An = Np, Pu, Am, and Cm) with different spin multiplicities at PBE0/B/6-311+G*/An/ECP60MWB, TPSSh/B/6-311+G*/An/ECP60MWB, and CCSD(T) levels; Figure S2: Molecular dynamics simulations of U@B_40_ (**1**) and U@B_40_^−^ (**2**) at 300 K, with the calculated root-mean-square-deviations (RMSDs) and maximum bond length deviations (MAXDs) indicated, respectively; Table S1: Optimized coordinates (x, y, z) of *C*_2*v*_ U@B_40_ (**1**), *C*_2*v*_ U@B_40_^−^ (**2**), *C*_2*v*_ Np@B_40_^+^ (**3**), *C*_2_ Np@B_40_ (**4**), *C*_2*v*_ Pu@B_40_ (**5**), *C*_2*v*_ Am@B_40_ (**6**), and *C*_2*v*_ Cm@B_40_^+^ (**7**) at the PBE0 level.
