# Supplementary material for: Investigation on the Coordination Bonding Nature of Actinide-Doped Endohedral Borospherenes An@B400/+/− (An = U, Np, Pu, Am, Cm)
Source: Molecules. 2024 Dec 13;29(24):5879. doi: 10.3390/molecules29245879 (PMC11677468; doi:10.3390/molecules29245879)
Supplement: Supplementary file 1 [file molecules-29-05879-s001.zip › molecules-3310619-supplementary.pdf]

Supporting Information

Investigation on the Coordination Bonding Nature of Actinide-Doped Endohedral Borospherenes $An@B_{40}^{0/+/-}$ (An = U, Np, Pu, Am, Cm)

Xiao-Ni Zhao, Zhi-Hong Wei and Si-Dian Li *

Key Laboratory of Chemical Biology and Molecular Engineering of Education Ministry, Institute of Molecular Science, Shanxi University, Taiyuan 030006, China; zhaoxiaoni@sxu.edu.cn (X.-N.Z.); weizhihong@sxu.edu.cn (Z.-H.W.)

* Correspondence: lisidian@sxu.edu.cn

Table of Contents

Figure S1. Relative energies of the low-lying isomers of $An@B_{40}^{0/+/-}$ (An = Np, Pu, Am and Cm) with different spin multiplicities at PBE0/B/6-311+G*/An/ECP60MWB, TPSSh/B/6-311+G*/An/ECP60MWB, and CCSD(T) levels.

Figure S2. Molecular dynamics simulations of $U@B_{40}$ (**1**) and $U@B_{40}^-$ (**2**) at 300 K, with the calculated root-mean-square-deviations (RMSD) and maximum bond length deviations (MAXD) indicated, respectively.

Table S1. Optimized coordinates (x, y, z) of C_{2v} $U@B_{40}$ **(1)**, C_{2v} $U@B_{40}^-$ **(2)**, C_{2v} $Np@B_{40}^+$ **(3)**, C_2 $Np@B_{40}$ **(4)**, C_{2v} $Pu@B_{40}$ **(5)**, C_{2v} $Am@B_{40}$ **(6)** and C_{2v} $Cm@B_{40}^+$ **(7)** at PBE0 level.

Figure S1. Relative energies (ΔE/eV) of the low-lying isomers of $An@B_{40}^{0/+/-}$ (An = Np to Cm) with different spin multiplicities at PBE0/B/6-311+G*/An/ECP60MWB, TPSSh/B/ 6-311+G*/An/ECP60MWB (in parentheses), and CCSD(T) (in square brackets) levels.

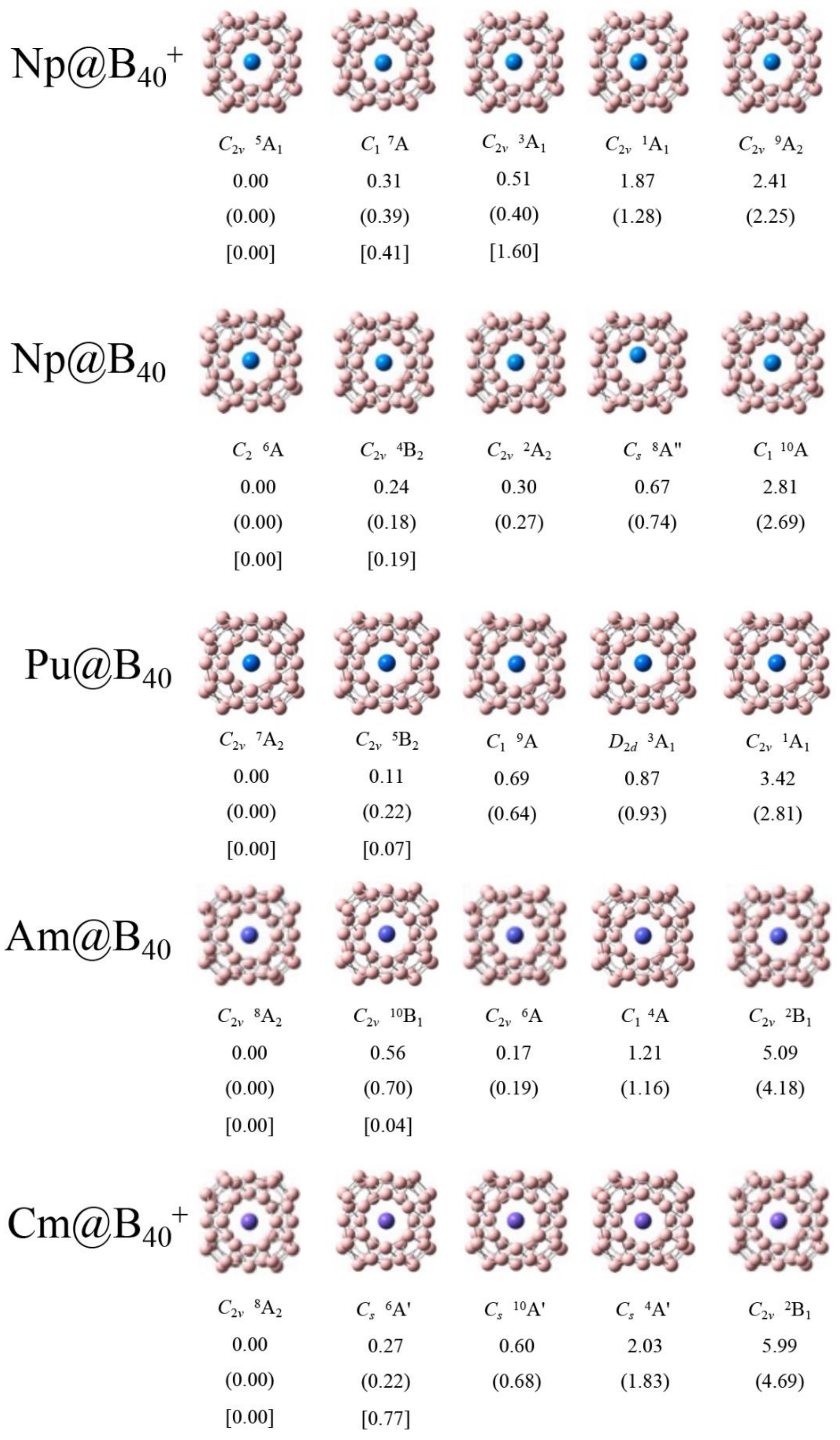

Figure S2. Molecular dynamics simulations of U@B_{40} (**1**) and U@B_{40}^- (**2**) at 300 K, with the calculated root-mean-square-deviations (RMSD/ Å) and maximum bond length deviations (MAXD/ Å) indicated, respectively.

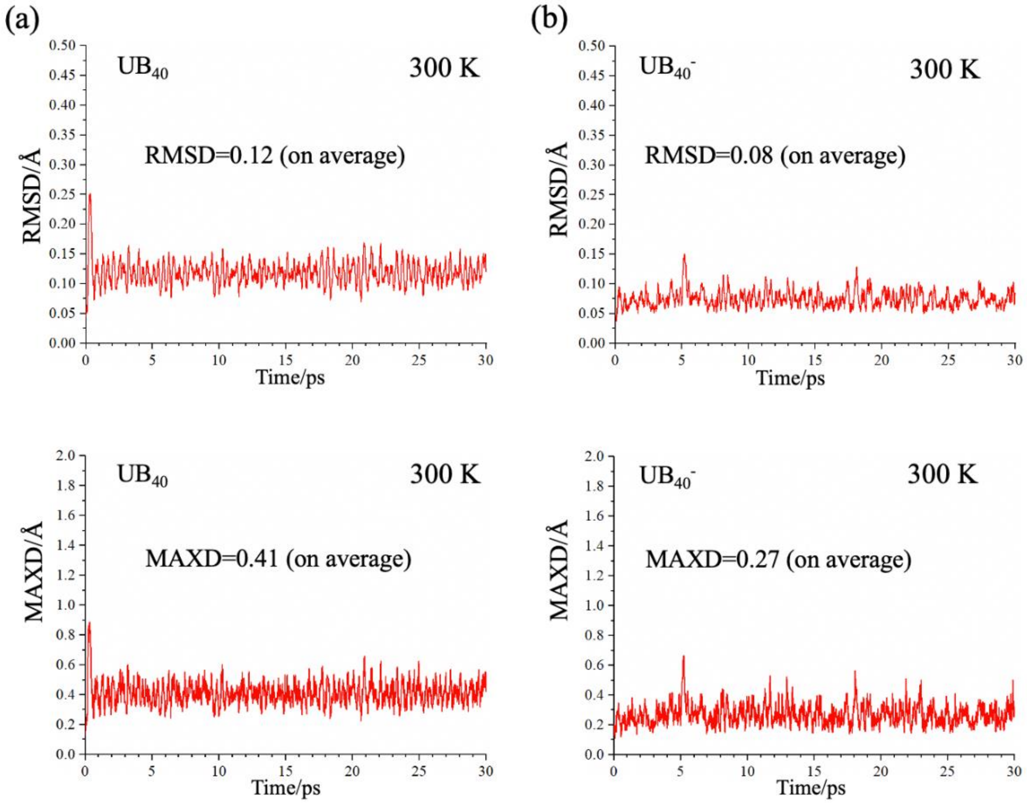

Table S1. Optimized coordinates (x, y, z) of C_{2v} U@B_{40} **(1)**, C_{2v} U@B_{40}^- **(2)**, C_{2v} Np@B_{40}^+ (**3**), C_2 Np@B_{40} (**4)**, C_{2v} Pu@B_{40} (**5)**, C_{2v} Am@B_{40} (**6**) and C_{2v} Cm@B_{40}^+ (**7**) at PBE0 level.

U@B_{40} (**1**, C_{2v}, $^3A_2$)

B	-2.69624900	1.64636400	-0.50927700
B	0.00000000	1.62191300	2.81038400
B	-2.40640000	1.39036100	1.12700800
B	2.69624900	-1.64636400	-0.50927700
B	1.71565000	0.00000000	-3.11263300
B	0.00000000	2.55775700	-2.08754800
B	0.87270600	-2.30420700	1.58541300
B	-1.41634700	-2.31056600	-1.36396900
B	-2.69624900	-1.64636400	-0.50927700
B	0.00000000	-1.62191300	2.81038400
B	-2.40640000	-1.39036100	1.12700800
B	1.65839600	2.61354300	0.25233500
B	2.40640000	1.39036100	1.12700800
B	2.40640000	-1.39036100	1.12700800
B	2.36017100	-0.84905600	-1.84287600
B	-2.36017100	0.84905600	-1.84287600
B	-0.87270600	-2.30420700	1.58541300
B	2.36017100	0.84905600	-1.84287600
B	2.70118900	0.00000000	1.87596900
B	-1.65839600	2.61354300	0.25233500
B	-0.87270600	2.30420700	1.58541300
B	0.87270600	2.30420700	1.58541300
B	1.41634700	-2.31056600	-1.36396900
B	-1.41634700	2.31056600	-1.36396900
B	1.41634700	2.31056600	-1.36396900
B	1.40163600	-0.85719700	2.49211000
B	1.40163600	0.85719700	2.49211000
B	2.69624900	1.64636400	-0.50927700
B	0.87799800	1.34147600	-2.74890700
B	0.00000000	-2.55775700	-2.08754800
B	-2.36017100	-0.84905600	-1.84287600
B	-1.40163600	-0.85719700	2.49211000
B	-1.40163600	0.85719700	2.49211000
B	-0.87799800	1.34147600	-2.74890700
B	1.65839600	-2.61354300	0.25233500
B	-2.70118900	0.00000000	1.87596900
B	0.87799800	-1.34147600	-2.74890700
B	-1.71565000	0.00000000	-3.11263300
B	-1.65839600	-2.61354300	0.25233500
B	-0.87799800	-1.34147600	-2.74890700
U	0.00000000	0.00000000	0.27501700

$U@B_{40}^{-}$ (**2**, C_{2v}, $^{4}B_{1}$)

B	-2.67666900	1.64198800	-0.54437500
B	0.00000000	1.62789300	2.79282700
B	-2.37730800	1.37070700	1.09293200
B	2.67666900	-1.64198800	-0.54437500
B	1.73266600	0.00000000	-3.17663900
B	0.00000000	2.55985200	-2.17786300
B	0.88360000	-2.31791900	1.57466900
B	-1.38262300	-2.28886200	-1.39093900
B	-2.67666900	-1.64198800	-0.54437500
B	0.00000000	-1.62789300	2.79282700
B	-2.37730800	-1.37070700	1.09293200
B	1.64453600	2.61493300	0.22742400
B	2.37730800	1.37070700	1.09293200
B	2.37730800	-1.37070700	1.09293200
B	2.34217200	-0.84665200	-1.87686100
B	-2.34217200	0.84665200	-1.87686100
B	-0.88360000	-2.31791900	1.57466900
B	2.34217200	0.84665200	-1.87686100
B	2.71610300	0.00000000	1.86512200
B	-1.64453600	2.61493300	0.22742400
B	-0.88360000	2.31791900	1.57466900
B	0.88360000	2.31791900	1.57466900
B	1.38262300	-2.28886200	-1.39093900
B	-1.38262300	2.28886200	-1.39093900
B	1.38262300	2.28886200	-1.39093900
B	1.40837600	-0.86080700	2.48239500
B	1.40837600	0.86080700	2.48239500
B	2.67666900	1.64198800	-0.54437500
B	0.88092700	1.33735400	-2.81868900
B	0.00000000	-2.55985200	-2.17786300
B	-2.34217200	-0.84665200	-1.87686100
B	-1.40837600	-0.86080700	2.48239500
B	-1.40837600	0.86080700	2.48239500
B	-0.88092700	1.33735400	-2.81868900
B	1.64453600	-2.61493300	0.22742400
B	-2.71610300	0.00000000	1.86512200
B	0.88092700	-1.33735400	-2.81868900
B	-1.73266600	0.00000000	-3.17663900
B	-1.64453600	-2.61493300	0.22742400
B	-0.88092700	-1.33735400	-2.81868900
U	0.00000000	0.00000000	0.34820000

NpB_{40}^{+} (**3**, C_{2v}, $^5A_1$)

B	-2.66787200	1.67840300	-0.47169800
B	0.00000000	1.63934200	2.85518800
B	-2.36227200	1.42910100	1.16258000
B	2.66787200	-1.67840300	-0.47169800
B	1.66348200	0.00000000	-3.03282000
B	0.00000000	2.61609200	-2.07681400
B	0.85513700	-2.32987600	1.60877500
B	-1.41146400	-2.36349000	-1.33742300
B	-2.66787200	-1.67840300	-0.47169800
B	0.00000000	-1.63934200	2.85518800
B	-2.36227200	-1.42910100	1.16258000
B	1.66908900	2.67266600	0.28154500
B	2.36227200	1.42910100	1.16258000
B	2.36227200	-1.42910100	1.16258000
B	2.32670000	-0.85232800	-1.78584300
B	-2.32670000	0.85232800	-1.78584300
B	-0.85513700	-2.32987600	1.60877500
B	2.32670000	0.85232800	-1.78584300
B	2.61427000	0.00000000	1.86225500
B	-1.66908900	2.67266600	0.28154500
B	-0.85513700	2.32987600	1.60877500
B	0.85513700	2.32987600	1.60877500
B	1.41146400	-2.36349000	-1.33742300
B	-1.41146400	2.36349000	-1.33742300
B	1.41146400	2.36349000	-1.33742300
B	1.38886600	-0.86936400	2.53415000
B	1.38886600	0.86936400	2.53415000
B	2.66787200	1.67840300	-0.47169800
B	0.86776900	1.38110600	-2.70932300
B	0.00000000	-2.61609200	-2.07681400
B	-2.32670000	-0.85232800	-1.78584300
B	-1.38886600	-0.86936400	2.53415000
B	-1.38886600	0.86936400	2.53415000
B	-0.86776900	1.38110600	-2.70932300
B	1.66908900	-2.67266600	0.28154500
B	-2.61427000	0.00000000	1.86225500
B	0.86776900	-1.38110600	-2.70932300
B	-1.66348200	0.00000000	-3.03282000
B	-1.66908900	-2.67266600	0.28154500
B	-0.86776900	-1.38110600	-2.70932300
Np	0.00000000	0.00000000	0.19641500

NpB_{40} (**4**, C_2, ^6A)

B	1.65064700	0.32538600	2.66032000
B	1.29937500	-2.98369700	0.00000000
B	1.29409400	-1.30612700	2.38187900
B	-1.66973800	0.68301800	-2.68131800
B	0.25748500	3.09823400	-1.71063600
B	2.71526900	1.87078900	0.00000000
B	-2.51022600	-1.33452200	-0.86879600
B	-2.23673900	1.59248800	1.38435900
B	-1.66973800	0.68301800	2.68131800
B	-1.94626200	-2.62433700	0.00000000
B	-1.53866200	-0.96236000	2.35039700
B	2.56193100	-0.52133100	-1.63830800
B	1.29409400	-1.30612700	-2.38187900
B	-1.53866200	-0.96236000	-2.35039700
B	-0.72654100	1.92698600	-2.34937300
B	0.96016900	1.70515000	2.30223400
B	-2.51022600	-1.33452200	0.86879600
B	0.96016900	1.70515000	-2.30223400
B	-0.21089300	-1.83232700	-2.63546600
B	2.56193100	-0.52133100	1.63830800
B	2.14397900	-1.84363300	0.87752200
B	2.14397900	-1.84363300	-0.87752200
B	-2.23673900	1.59248800	-1.38435900
B	2.39450000	1.13587200	1.40262600
B	2.39450000	1.13587200	-1.40262600
B	-1.14530300	-2.40088400	-1.40203400
B	0.58695700	-2.59424800	-1.39739000
B	1.65064700	0.32538600	-2.66032000
B	1.57130600	2.63763100	-0.88587200
B	-2.43627600	2.39077400	0.00000000
B	-0.72654100	1.92698600	2.34937300
B	-1.14530300	-2.40088400	1.40203400
B	0.58695700	-2.59424800	1.39739000
B	1.57130600	2.63763100	0.88587200
B	-2.72437500	0.01454200	-1.67708700
B	-0.21089300	-1.83232700	2.63546600
B	-1.12833900	2.85942900	-0.87510000
B	0.25748500	3.09823400	1.71063600
B	-2.72437500	0.01454200	1.67708700
B	-1.12833900	2.85942900	0.87510000
Np	0.07029000	-0.26990000	0.00000000

PuB_{40} (**5**, C_{2v}, $^7A_2$)

B	-2.64681000	1.66314400	-0.53125400
B	0.00000000	1.63344800	2.81511500
B	-2.35927200	1.42345900	1.11326300
B	2.64681000	-1.66314400	-0.53125400
B	1.68748900	0.00000000	-3.12760700
B	0.00000000	2.61887900	-2.19403600
B	0.86247100	-2.33565000	1.57466900
B	-1.38066500	-2.33844900	-1.39971100
B	-2.64681000	-1.66314400	-0.53125400
B	0.00000000	-1.63344800	2.81511500
B	-2.35927200	-1.42345900	1.11326300
B	1.64719500	2.65439900	0.22702300
B	2.35927200	1.42345900	1.11326300
B	2.35927200	-1.42345900	1.11326300
B	2.31360600	-0.85254300	-1.85887300
B	-2.31360600	0.85254300	-1.85887300
B	-0.86247100	-2.33565000	1.57466900
B	2.31360600	0.85254300	-1.85887300
B	2.62692100	0.00000000	1.82160000
B	-1.64719500	2.65439900	0.22702300
B	-0.86247100	2.33565000	1.57466900
B	0.86247100	2.33565000	1.57466900
B	1.38066500	-2.33844900	-1.39971100
B	-1.38066500	2.33844900	-1.39971100
B	1.38066500	2.33844900	-1.39971100
B	1.40254300	-0.87522200	2.50631600
B	1.40254300	0.87522200	2.50631600
B	2.64681000	1.66314400	-0.53125400
B	0.87562800	1.37558400	-2.79653600
B	0.00000000	-2.61887900	-2.19403600
B	-2.31360600	-0.85254300	-1.85887300
B	-1.40254300	-0.87522200	2.50631600
B	-1.40254300	0.87522200	2.50631600
B	-0.87562800	1.37558400	-2.79653600
B	1.64719500	-2.65439900	0.22702300
B	-2.62692100	0.00000000	1.82160000
B	0.87562800	-1.37558400	-2.79653600
B	-1.68748900	0.00000000	-3.12760700
B	-1.64719500	-2.65439900	0.22702300
B	-0.87562800	-1.37558400	-2.79653600
Pu	0.00000000	0.00000000	0.32075900

AmB_{40}(**6**, C_{2v}, $^8A_2$)

B	2.65580200	1.65094800	0.23288700
B	0.00000000	1.68530100	-3.12771500
B	2.34504600	1.38942700	-1.39959700
B	-2.65580200	-1.65094800	0.23288700
B	-1.63684400	0.00000000	2.82231000
B	0.00000000	2.62506200	1.83143500
B	-0.85082500	-2.31133600	-1.85373400
B	1.42857800	-2.36523300	1.12123600
B	2.65580200	-1.65094800	0.23288700
B	0.00000000	-1.68530100	-3.12771500
B	2.34504600	-1.38942700	-1.39959700
B	-1.66635900	2.64680900	-0.52866100
B	-2.34504600	1.38942700	-1.39959700
B	-2.34504600	-1.38942700	-1.39959700
B	-2.34052000	-0.86197100	1.58169900
B	2.34052000	0.86197100	1.58169900
B	0.85082500	-2.31133600	-1.85373400
B	-2.34052000	0.86197100	1.58169900
B	-2.61831300	0.00000000	-2.18115300
B	1.66635900	2.64680900	-0.52866100
B	0.85082500	2.31133600	-1.85373400
B	-0.85082500	2.31133600	-1.85373400
B	-1.42857800	-2.36523300	1.12123600
B	1.42857800	2.36523300	1.12123600
B	-1.42857800	2.36523300	1.12123600
B	-1.37493800	-0.87189400	-2.79253300
B	-1.37493800	0.87189400	-2.79253300
B	-2.65580200	1.65094800	0.23288700
B	-0.87610700	1.40236700	2.51205400
B	0.00000000	-2.62506200	1.83143500
B	2.34052000	-0.86197100	1.58169900
B	1.37493800	-0.87189400	-2.79253300
B	1.37493800	0.87189400	-2.79253300
B	0.87610700	1.40236700	2.51205400
B	-1.66635900	-2.64680900	-0.52866100
B	2.61831300	0.00000000	-2.18115300
B	-0.87610700	-1.40236700	2.51205400
B	1.63684400	0.00000000	2.82231000
B	1.66635900	-2.64680900	-0.52866100
B	0.87610700	-1.40236700	2.51205400
Am	0.00000000	0.00000000	0.30614900

CmB_{40}^{+} (**7**, C_{2v}, $^8A_2$)

B	2.66022600	1.65896800	0.26462500
B	0.00000000	1.66481900	-3.07008900
B	2.36452100	1.40811300	-1.36627500
B	-2.66022600	-1.65896800	0.26462500
B	-1.63370700	0.00000000	2.84908400
B	0.00000000	2.62175000	1.85621900
B	-0.85069200	-2.31721900	-1.81368000
B	1.43434900	-2.36940100	1.15398200
B	2.66022600	-1.65896800	0.26462500
B	0.00000000	-1.66481900	-3.07008900
B	2.36452100	-1.40811300	-1.36627500
B	-1.67072100	2.65498900	-0.49323900
B	-2.36452100	1.40811300	-1.36627500
B	-2.36452100	-1.40811300	-1.36627500
B	-2.33038400	-0.85809600	1.60462100
B	2.33038400	0.85809600	1.60462100
B	0.85069200	-2.31721900	-1.81368000
B	-2.33038400	0.85809600	1.60462100
B	-2.62305800	0.00000000	-2.11520400
B	1.67072100	2.65498900	-0.49323900
B	0.85069200	2.31721900	-1.81368000
B	-0.85069200	2.31721900	-1.81368000
B	-1.43434900	-2.36940100	1.15398200
B	1.43434900	2.36940100	1.15398200
B	-1.43434900	2.36940100	1.15398200
B	-1.38340900	-0.86795100	-2.74124900
B	-1.38340900	0.86795100	-2.74124900
B	-2.66022600	1.65896800	0.26462500
B	-0.87083000	1.39680100	2.53126100
B	0.00000000	-2.62175000	1.85621900
B	2.33038400	-0.85809600	1.60462100
B	1.38340900	-0.86795100	-2.74124900
B	1.38340900	0.86795100	-2.74124900
B	0.87083000	1.39680100	2.53126100
B	-1.67072100	-2.65498900	-0.49323900
B	2.62305800	0.00000000	-2.11520400
B	-0.87083000	-1.39680100	2.53126100
B	1.63370700	0.00000000	2.84908400
B	1.67072100	-2.65498900	-0.49323900
B	0.87083000	-1.39680100	2.53126100
Cm	0.00000000	0.00000000	0.22915600
